# Na/K-ATPase and Regulation of Sperm Function

**DOI:** 10.21451/1984-3143-AR2018-0024

**Published:** 2018-08-03

**Authors:** Jacob C. Thundathil, Gayathri D. Rajamanickam, John P Kastelic

**Affiliations:** 1 Department of Production Animal Health, Faculty of Veterinary Medicine, University of Calgary, Calgary, Alberta, Canada; 2 Department of Veterinary Clinical and Diagnostic Services, Faculty of Veterinary Medicine, University of Calgary, Calgary, Alberta, Canada

**Keywords:** bull sperm, Na/K-ATPase, ouabain.

## Abstract

A standard bull breeding soundness evaluation (BBSE) identifies bulls with semen that is grossly abnormal. Nonetheless, semen samples classified as satisfactory based on these traditional approaches differ in fertility; perhaps there are submicroscopic differences in sperm characteristics affecting fertility. Therefore, a better understanding of molecular regulation of sperm function could promote development of novel, evidence-based approaches to predict male fertility. Recently the α4 isoform of Na/K-ATPase (ATP1A4) has received considerable attention, due to its testis- specific expression in post-meiotic germ cells and mature sperm, in addition to its regulation of sperm motility and capacitation. Using fresh bull sperm, we determined that ATP1A4 resided in specialized microdomains (raft and non-raft) of the sperm plasma membrane and activated specific signaling (caveolin-1, EGFR, Src, ERK1/2) molecules during sperm capacitation. Furthermore, ATP1A4 was the predominant isoform responsible for total Na/K-ATPase activity in capacitated sperm. Despite the widely accepted dogma of transcriptional/translational quiescence, bovine sperm translated ATP1A4 mRNA on mitochondrial or mitochondrial-type ribosomes, increasing their content and activity during capacitation. Proteomic analysis of raft and non-raft fractions revealed a significant interaction between ATP1A4 and plakoglobin, a member of the β-catenin family of proteins involved in cell adhesion, in the equatorial segment of capacitated sperm, suggesting a potential role in sperm-oolemma fusion. In frozen-thawed sperm, ATP1A4 content and activity was greater in high- versus low-fertility bulls. Additionally, ATP1A4-induced increases in ROS, calcium, actin polymerization and tyrosine phosphorylation were also involved in regulating post-thaw sperm function in these bulls. Overall, results demonstrated that ATP1A4 had unique roles in controlling several aspects of sperm physiology, acting through well-established enzyme activity and signaling functions. Consequently, isoforms of Na/K-ATPase are potential biomarkers for male fertility.

## Introduction to bull fertility evaluation

Dairy and beef industries strive to achieve high pregnancy rates from genetically superior bulls. Therefore, fertility is more important than production traits. Based on the assessment from the American Gelbvieh Association’s Alliance marketing program on 110,000 feedlot cattle, estimated relative importance of reproductive traits to growth and carcass traits are in the ratio of 4:2:1, respectively (Schiefelbein, 1998). Bulls with reduced sperm fertility can cause substantial economic losses due to delayed conception, prolonged calving seasons, reduced calf weaning weights, and increased number of breeding females that are culled due to either failure to become pregnant or to delayed pregnancy establishment. Although subfertility of beef bulls may not be evident when used in a multiple-sire or low breeding pressure system, such bulls typically have reduced fertility when they are used for single- sire mating or AI (Kastelic and Thundathil, 2008; Kasimanickam *et al*., 2012). Therefore, bull effects are of utmost importance. For example, a modest 1% increase in the reproductive rate would generate up to three times more return on investments (Hansen, 2006). Due to the subjective nature of conventional semen analysis, acceptable semen may be erroneously rejected, and concurrently, semen of unacceptable quality may be used for inseminations (Christensen *et al*., 2005). Therefore, in addition to frozen semen evaluation, bull or semen selection could be complemented by laboratory assays focused on molecular sperm function.

## Role of biomarkers in bull fertility evaluation

High-throughput technologies such as mass spectrometry, along with appropriate bioinformatics tools, may provide better cues for annotation of proteins in the context of gamete biology. As proteins define a cell phenotype, changes at the proteome level could lead to differences in phenotypes influencing economically important traits and could enable identification of biomarkers of fertility. For example, content of P25b, a bovine sperm membrane antigen, was lower in semen from subfertile bulls than in high-fertility bulls (Parent *et al*., 1999). A 30-kDa heparin-binding protein (fertility-associated antigen, FAA), was differentially expressed in sperm membranes of beef bulls with varying fertility (Bellin *et al*., 1998). Furthermore, Sutovsky *et al*. (2015) described positive and negative protein biomarkers of fertility. Negative fertility markers include proteins that are exclusively associated with certain types of sperm defects, whereas positive biomarkers include proteins that are present in normal sperm, although they may either be upregulated or downregulated. One of the negative protein biomarkers of sperm quality is ubiquitin, which has been assessed in numerous species, including humans (Sutovsky *et al*., 2001) horses (Sutovsky *et al*., 2003), cattle (Sutovsky *et al*., 2002), and pigs (Kuster *et al*., 2004), and is correlated with infertility and indications of poor sperm quality, namely primary and total morphological defects (Purdy, 2008). Comparing normal versus abnormal sperm induced by elevated testicular temperature, Newton *et al*. (2009) demonstrated differential expression of several sperm proteins in morphologically abnormal sperm, including the testis-specific isoform of Na/K-ATPase (ATP1A4), as the molecular basis for impaired function.

## Na/K-ATPase structure and isoforms

Na/K-ATPase is a plasma membrane protein with two fundamental roles in regulation of cell function. First, it is responsible for maintenance of Na^+^ and K^+^ gradients across the plasma membrane of most mammalian cells. In that regard, this enzyme is responsible for cell volume and pH, resting membrane potential, osmotic balance, and generation of a Na^+^ gradient essential for secondary transmembrane ion transport (Skou and Esmann, 1992). Secondly, it acts as a receptor for cardiotonic steroids such as ouabain (specific inhibitor of Na/K-ATPase) and its interaction initiates signaling critical for regulation of various cell functions. The functional Na/K-ATPase consists of two subunits, the α subunit (110 kDa) and the β subunit (35-60 kDa, depending on glycosylation; (Blanco and Mercer, 1998). The α polypeptide is the catalytic unit responsible for ionic translocation as well as ouabain- dependent signaling events (Jorgensen *et al*., 2003), whereas the β subunit is essential for the enzyme’s activity, as well as folding and localisation in the membrane (Geering, 1991). There are four α isoforms (α1, α2, α3, and α4) and three β isoforms (β1, β2, and β3) that are expressed in mammalian tissues (Blanco and Mercer, 1998; Mobasheri *et al*., 2000). The α1 and β1 isoforms are expressed in almost every cell (function as housekeeping Na/K-ATPase), whereas other α polypeptides have more restricted expressions, with specific roles (Mobasheri *et al*., 2000).

Ouabain in secreted from the adrenal gland (Doris *et al*., 1984) and is also identified in the bovine vaginal fluid (Daniel *et al*., 2010). Therefore, the presence of Na/K-ATPase in sperm and ouabain in the female reproductive tract suggests that this protein has a specific role in sperm physiology during capacitation or in other steps leading to fertilization.

## Expression and localization of Na/K-ATPase isoforms in testis and sperm

The Na/K-ATPase α4 isoform (ATP1A4) has received considerable attention in recent years due to its sperm-specific expression (Blanco *et al*., 2000). The Na/K-ATPase α1 (ATP1A1; ubiquitous isoform) and α4 subunits are co-expressed in sperm with the β1 and β3 isoforms (Arystarkhova and Sweadner, 1997). In addition to α1, α4, β1, and β3 isoforms, the α3 and β2 subunits are also present in bovine sperm (Hickey and Buhr, 2011). In sperm two-thirds of total Na/K-ATPase activity is attributed to ATP1A4 (Wagoner *et al*., 2005). ATP1A4 expression peaked in mature testes in rats (Woo *et al*., 2000; Wagoner *et al*., 2005), whereas ATP1A1 expression was constant throughout spermatogenesis. In terms of localization, ATP1A4 was restricted to the mid-piece (rat) and principal piece (human; (Woo *et al*., 2000; Hlivko *et al*., 2006) of the flagellum, whereas ATP1A1 was present throughout the flagellum (Wagoner *et al*., 2005). In total contrast, in our studies with fresh bovine sperm, ATP1A4 was restricted to the sperm head (Thundathil *et al*., 2006).

## Ion transport-dependent functions of Na/K-ATPase in sperm

An isoform of Na/K-ATPase (ATP1A4) specifically expressed in sperm suggests that this protein has a specific role in sperm physiology. Consequently, it was no surprise that sperm from ATP1A4 KO mice demonstrated severe reduction in total motility due to a characteristic bend in the sperm tail and cell membrane depolarization (Jimenez *et al*., 2011a). Simultaneously, over-expression of ATP1A4 resulted in plasma membrane hyperpolarization, higher progressive motility and enhanced hyperactivation, implicating ATP1A4 in sperm motility under both noncapacitating and capacitating conditions (Jimenez *et al*., 2011b). In addition, Jimenez *et al*. (2012) also reported that ATP1A4 activity was upregulated at the plasma membrane during sperm capacitation. ATP1A4 indirectly influences sperm motility by regulating intracellular pH, membrane potential and intracellular calcium release. ATP1A4 is linked to other K^+^ channels which are involved in depolarization and hyperpolarization. Consequently, ouabain inhibition of ATP1A4 caused sperm membrane depolarization (Jimenez *et al*., 2012). ATP1A4 is also coupled to Na/H-exchanger (NHE), a flagellar protein, that uses the Na^+^ gradient established by the Na/K-ATPase to remove H^+^ from the cell in exchange for Na^+^ (Counillon and Pouyssegur, 2000), thereby regulating the intracellular pH. Therefore, inhibition of ATP1A4 by ouabain eliminates the Na^+^ gradient used by NHE to move H^+^ out of the cell. Loss of NHE activity may lead to acidification of the intracellular compartment, which abolishes dynein activity and reduces flagellar movement (Woo *et al*., 2002). Furthermore, ATP1A4 is also functionally linked to sperm calcium regulation via a Na/Ca-exchanger (NCX), which uses the secondary Na^+^ influx generated by Na/K-ATPase for calcium efflux. Inhibition of Na/K-ATPase increases intracellular Na^+^ concentration which disrupts calcium efflux by NCX, thereby increasing intracellular calcium concentrations (Jimenez *et al*., 2010).

## Na/K-ATPase as a signaling molecule during bovine sperm capacitation

Based on known signaling roles of Na/K- ATPase in somatic cells, ouabain (Na/K-ATPase inhibitor) induces ERK1/2 activation via transactivation of EGFR and activation of PLC and PKC. In addition, Na/K-ATPase-ouabain interaction generates ROS from mitochondria through the Src/EGFR/Ras pathway (Liu *et al*. 2000; Xie *et al*., 1999). Therefore, the sequalae of Na/K-ATPase signaling events in somatic cells resemble some events associated with sperm capacitation, namely increases in intracellular Na^+^ and Ca^2+^ concentrations, generation of ROS, and activation of ERK1/2. Protein kinase A (PKA) mediated activation of protein tyrosine kinase (PTK) induces tyrosine phosphorylation (a hallmark of capacitation) in sperm (de Lamirande and O’Flaherty, 2008). There is also cross-talk between ROS and elements of ERK1/2, PKA and PTK (O’Flaherty *et al*., 2006) to promote tyrosine phosphorylation during capacitation. Therefore, we tested involvement of these signaling molecules in bovine sperm and demonstrated that PKA, RTK and Src kinases are involved in this process (Newton *et al*., 2010). Another study in bovine sperm also confirmed involvement of the ERK pathway in this process (Anpalakan, 2010).

Our previous study (Newton *et al*., 2010) demonstrated that incubation of bovine sperm with a PKA inhibitor (H89) inhibited ouabain-induced tyrosine phosphorylation. In addition, activated PKA interacts with PKC and activates phospholipase D (PLD), which subsequently hydrolyses phosphatidyl choline (PC) to phosphatidic acid (PA), mediating polymerization of globular (G)-actin to filamentous (F)-actin. Actin polymerization is involved in capacitation and the acrosome reaction in bovine sperm (Yagi and Paranko, 1995; Cohen *et al*., 2004). However, mechanisms by which ATP1A4 regulates these multiple signaling pathways during capacitation remain unknown. We proposed that raft and non-raft pools of ATP1A4 exist in sperm membrane and activate specific signaling molecules/pathways (investigated in studies reported below).

## Role of lipid rafts and non-rafts in ATP1A4 signaling during bovine sperm capacitation

In somatic cells, the majority of Na/K-ATPase resides in specialized microdomains of the plasma membrane called lipid rafts, which facilitates its signaling functions, due to proximity to other signaling molecules within these microdomains (Liu *et al*, 2003; Liang *et al*, 2007). Domains similar to somatic cell lipid rafts have been demonstrated in mammalian sperm (Cross, 2004; Shadan *et al*., 2004; Sleight *et al*., 2005; Bou Khalil *et al*., 2006; Nixon and Aitken, 2009) and several raft subtypes have also been identified (Asano *et al*, 2009). Although previous studies demonstrated a role for lipid raft proteins in sperm-oocyte interactions (van Gestel *et al*., 2005; van Gestel *et al*., 2007), involvement of raft and non-raft proteins in signaling events leading to sperm capacitation has apparently not been reported. In our recent study (Rajamanickam *et al*., 2017a), content of ATP1A4 was increased in both raft and non-raft fractions of capacitated sperm.

In addition, ATP1A4 was the predominant isoform contributing to total Na/K-ATPase activity in both rafts and non-rafts of capacitated sperm. With reference to signaling functions of ATP1A4, there were comparative increases in phosphorylation of signaling molecules in both raft (Cav1) and non-raft (EGFR, Src, and ERK1/2) membrane fractions during capacitation. We also inferred that ATP1A4 interacted with Cav1 and EGFR in the raft fraction, whereas interactions of ATP1A4 with Src, EGFR, and ERK1/2 occurred in the non-raft fraction of ouabain-capacitated sperm. Therefore, we concluded that both raft and non-raft cohorts of ATP1A4 contributed to enzyme activity and phosphorylation of signaling molecules during bovine sperm capacitation.

**Figure 1:**
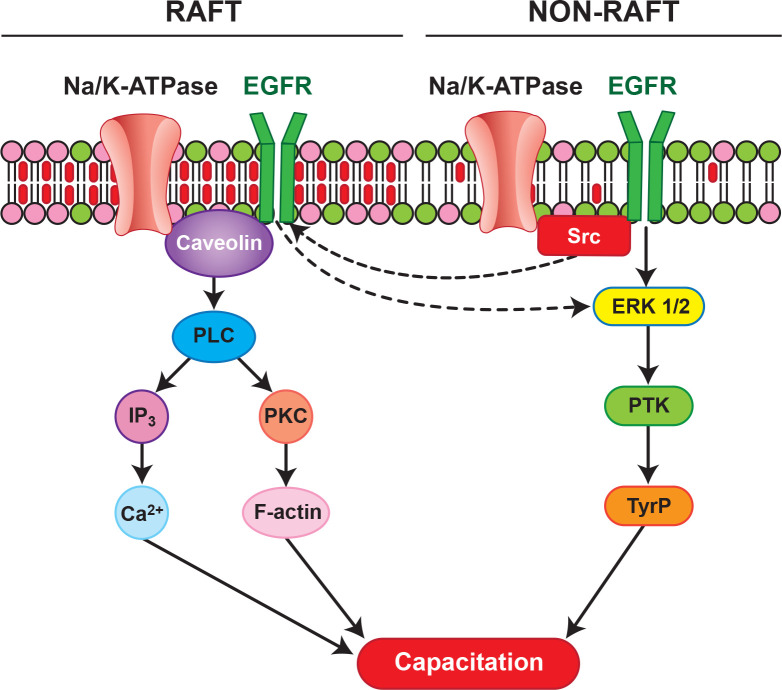
A hypothetical model for ATP1A4-mediated raft and non-raft signaling pathways during bovine sperm capacitation. Interactions between Na/K-ATPase isoform and Cav1, can activate phospholipase C (PLC), thereby generating inositol 1,4,5-triphosphate (IP_3_) and diacyl glycerol (DAG), via hydrolysis of phosphatidylinositol 4,5- bisphosphate which in turn activates protein kinase C (PKC). IP_3_ binds to its cognate receptor (IP_3_R), leading to an increase in intracellular calcium, whereas PKC mediates conversion of globular actin (G-actin) to filamentous-actin (F-actin) through other mediator proteins. Within non-rafts, Na/K-ATPase-ouabain interaction involves Src and EGFR. Signals from non-raft interactions are relayed downstream, leading to activation of ERK1/2 and protein tyrosine kinase (PTK)-mediated tyrosine phosphorylation of proteins. Increase in F-actin, intracellular calcium, and protein tyrosine phosphorylation contribute to capacitation-associated changes in sperm

**Figure 2:**
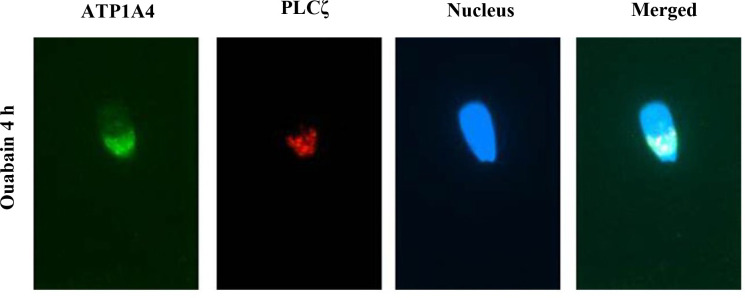
Co-localisation of ATP1A4-PLCζ during sperm capacitation. Representative images of ATP1A4 (green), PLCζ (red), nuclei (blue) and merged ATP1A4, PLCζ and DAPI staining in ouabain-capacitated sperm.

We proposed a model regarding potential raft- and non-raft-mediated ATP1A4 signaling pathways (Fig. 1) in the bovine sperm plasma membrane during capacitation. Upstream events in rafts may involve interactions between Na/K-ATPase isoforms and CAV1, which can bind and activate Phospholipase C (PLC), thereby increasing hydrolysis of phosphatidylinositol 4,5-bisphosphate (PIP_2_), generating inositol 1,4,5-triphosphate (IP_3_) and diacyl glycerol (DAG), which in turn activate protein kinase C (PKC). Co-localization of ATP1A4 and PLC isoform (PLCζ) was performed to determine involvement of PLCζ in our raft signaling model. PLCζ was localized to the equatorial segment (ES) and ATP1A4 co-localised with PLCζ at the ES and postacrosome region in ouabain- capacitated sperm (Fig. 2). IP_3_ binds to its cognate receptor (IP_3_R), leading to an increase in intracellular calcium, whereas PKC through other mediator proteins promotes polymerization of globular actin (G-actin) to filamentous-actin (F-actin). Within non-rafts, ATP1A4- ouabain interaction involves Src and EGFR leading to activation of ERK1/2 and protein tyrosine kinase (PTK)-mediated tyrosine phosphorylation of proteins. Increases in F-actin, intracellular calcium, and protein tyrosine phosphorylation contribute to capacitation- associated changes in sperm. The above-mentioned model assumes that activation/phosphorylation of signaling molecules occurs within the respective domains (raft or non-raft) of the plasma membrane. However, it is likely that a subset of the non-raft pool of ATP1A4 merges with the raft pool of ATP1A4 (or vice- versa), due to cholesterol efflux, either amplifying the existing signaling process or initiating a new downstream pathway during capacitation. All these possibilities open exciting areas for future investigation and could unravel the dynamic organization of signaling complexes mediated by ATP1A4 in sperm.

## Characterization of the ATP1A4 interactome in lipid rafts and non-rafts during bovine sperm capacitation

In the aforementioned study, we highlighted two distinct pools (raft and non-raft) of ATP1A4 that trigger specific downstream signaling pathways. However, during spermatogenesis, sperm acquire several unique proteins or their isoforms to meet its functional demands, including lactate dehydrogenase (LDH-C4), sperm adhesion molecule 1 (PH-20), and testis-specific isoform of Angiotensin Converting Enzyme (t-ACE). Therefore, it is likely that ATP1A4 interacts with other sperm proteins during capacitation. To test this, we used an immunoprecipitation-mass spectrometry approach to compare the interactome profile of ATP1A4 between raft- and non-raft membrane fractions from capacitated sperm.

**Figure 3:**
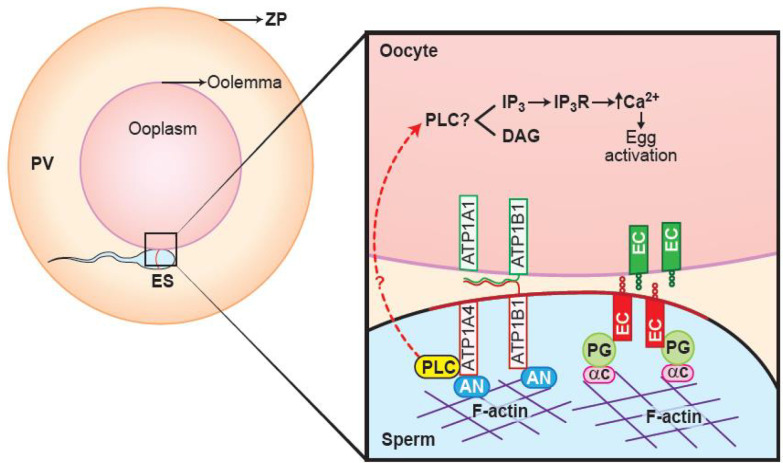
A hypothetical model depicting the involvement of plakoglobin, α and β subunits of ATP1A4, E-cadherin and PLCζ during sperm-oocyte fusion. Complementary E-cadherin molecules on the sperm and oocyte could bind to each other; these interactions could be further strengthened by binding of the cytoplasmic domain of E-cadherin (EC) to plakoglobin (PG) -α-catenin (αC) -actin network. ATP1A4 could bind to an anchor protein, ankyrin (AN), which in turn interacts with the actin cytoskeleton and indirect links ATP1A4 to plakoglobin-E-cadherin complex. This interaction could facilitate entry of sperm-derived PLCζ (indicated by broken arrows) which results in increase of intracellular calcium and resumption of meiosis in the oocyte. In addition, ATP1A4 could be involved in sperm- oocyte interactions by N-glycan motifs-mediated binding of β-subunits of Na/K-ATPase (ATP1B1/ATP1B3) present in gametes.

Proteomic analysis using a gel-based LC- MS/MS approach identified that the non-raft interactome was comprised of hexokinase, plakophilin- 1, 14-3-3 protein, cathepsin D, and heat shock protein. A disintegrin and metalloprotease (ADAM) and annexin A2 were exclusive to the raft fraction, whereas actin and plakoglobin were identified in both raft and non-raft fractions of ouabain-capacitated sperm. These differentially interacted proteins are putatively involved in sperm-oocyte interactions, metabolism, and protease activity and they also act as adaptor and cytoskeletal proteins, based on gene ontological information. We validated plakoglobin, among other proteins, due to its significant interaction with ATP1A4. Immunocytochemical staining demonstrated that plakoglobin was localized to the equatorial segment of uncapacitated and capacitated sperm. The ATP1A4 signal was predominantly localized to the anterior acrosome in uncapacitated sperm, became pronounced in the equatorial segment, and co-localized with plakoglobin in capacitated sperm (Fig. 3).

In epithelial cells, E-cadherin, catenins and the actin cytoskeleton mediate cell-cell adhesion (Yamada *et al*., 2005). In addition, E-cadherin and catenin proteins are also expressed in sperm and oocytes (Rufas *et al*., 2000; DeVries *et al*., 2004) where they are specifically localized to the equatorial segment of the sperm head and microvillar region on oolemma (Takezawa *et al*., 2011). If gamete interaction involves mechanisms that are like epithelial cell-cell adhesion, it is likely that E-cadherin and catenin are involved in events leading to sperm-oocyte adhesion and fusion, considering their strategic locations in sperm and in oocytes. Since plakoglobin belongs to the catenin family, interaction between ATP1A4 and plakoglobin could potentially mediate gamete fusion, leading to fertilization. However, the physiological relevance of Na/K-ATPase-plakoglobin interactions remains to be investigated.

## Capacitation increases the content of ATP1A4 in bovine sperm via mitochondrial translation

Despite the widely accepted dogma of transcriptional/translational quiescence in sperm, we observed that increased ATP1A4 content in raft and non-raft membrane fractions during capacitation with either ouabain or heparin. This increase in ATP1A4 content in the plasma membrane was further confirmed with immunoblotting using total sperm protein extracts, flow cytometry in fixed capacitated sperm, and a simultaneous increase in the enzyme activity of the protein. Furthermore, the increase in content and activity were not due to relocation of this protein to plasma membrane from other subcellular components. Upon further investigation, we also identified that this capacitation-associated increase was partially sensitive to the mitochondrial translation inhibitor chloramphenicol but insensitive to actinomycin D (a transcription inhibitor), suggesting mitochondrial translation of ATP1A4 during capacitation.

Ejaculated sperm contain a complex repertoire of transcripts and intact mRNAs (Sendler *et al*., 2013; Das *et al*., 2013). Furthermore, subcellular localization of mRNA in sperm has been documented (Kumar *et al*., 1993). It was suggested that *de novo* protein translation from sperm mRNA is essential to supplement degraded proteins or to support functional changes during capacitation. This *de novo* protein production was also demonstrated in other mammals, suggesting widespread existence of this mechanism across species (Gur and Breitbart 2006). Mitochondrial ribosomes seem to be responsible for *de novo* protein synthesis during capacitation as sperm lacks classical cytoplasmic machinery (Ostermeier *et al*., 2002; Gilbert *et al*., 2007). Accordingly, several publications have highlighted that cycloheximide (cytoplasmic ribosome inhibitor) did not affect sperm protein synthesis (Bragg and Handel 1979; Ahmed *et al*., 1984; Naz 1998; Gur and Breitbart 2006).

**Figure 4:**
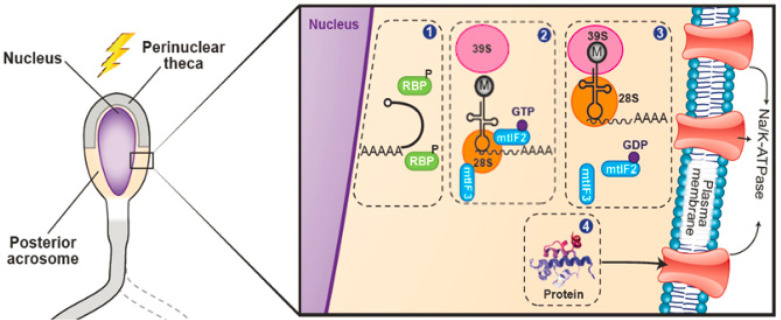
Hypothetical schematic view of the possible mechanisms involved in translation of sperm mRNA during capacitation. During spermatogenesis, RBPs bind with pre-mRNA in the nucleus and are subsequently exported together to the cytoplasm of spermatocytes and early round spermatids. The perinuclear theca (PT) and postacrosomal sheath (PS) in the sperm could serve as translation sites in the sperm head. (1) During capacitation, post-translational protein modifications such as phosphorylation, release the mRNA from the inhibitory effect of RBPs. (2) Mitochondrial translation initiation factors, mtIF3 and mtIF2 facilitate binding of the mRNA and tRNA^Met^, respectively, to 28S (small mitochondrial ribosome subunit) in the presence of GTP hydrolysis. (3) Molecular events described in Step 2 initiate binding of 39S (large mitochondrial ribosome subunit) to 28S-mRNA-tRNA complex and formation of a functional 55S mitochondrial or mitochondrial-type ribosome. (4) Synthesis of ATP1A4 protein and its translocation to the plasma membrane.

A standard assay to demonstrate protein synthesis in any cell type is incorporation of labeled amino acids into newly synthesized peptides. In our study, bodipy-tagged lysine was incorporated into several proteins at ∼110, 75, 50 and 37 kDa; however, in the presence of chloramphenicol (mitochondrial translation inhibitor), band intensities at ∼110 and 37 kDa were partially inhibited, whereas bands at 75 and 50 kDa were completely abolished, suggesting that protein synthesis occurred during bovine sperm capacitation. Mitochondrial or mitochondrial-type ribosomes may perform a similar function in sperm, as cytoplasmic machinery is lost during spermatogenesis (Toshimori, 2009). Interestingly, extra-mitochondrial localization of mitochondrial ribosomes, especially the 16S rRNA, has been demonstrated in mouse oocytes and zygotes (Ninomiya and Ichinose, 2007) and sperm nucleus (Villegas *et al*., 2002) and functional competence of such mitochondrial ribosomes has been demonstrated in *Drosophila* embryos (Amikura *et al*., 2001; Amikura *et al*., 2005). In the current model (Fig. 4), we hypothesize that ATP1A4 mRNA, mitochondrial or mitochondrial-type ribosomes and translation initiation and elongation factors are present outside mitochondria (perinuclear theca and post-acrosome regions in the head) in ejaculated sperm. However, ATP1A4 mRNA is prevented from being translated due to the inhibitory action of RBPs (RNA binding proteins) and the unavailability of a functional 55 S mitochondrial ribosome in ejaculated sperm. Capacitation associated changes remove the inhibitory effect of RBPs and the binding of the mRNA and tRNA^Met^ to the small ribosome subunit (28 S). The latter step is markedly enhanced in the presence of GTP. These initial steps recruit the large ribosome subunit (39 S) to join the small ribosome subunit (28 S) and thereby a fully functional mitochondrial ribsome is assembled. The initiation phase is completed, and translation can proceed with elongation and termination phases leading to production of a functional protein (Rajamanickam *et al*., 2017c).

Perhaps post-translational modifications of ATP1A4 are carried out through hydrophobic and electrostatic interactions of membrane-associated molecular chaperones ultimately guiding them to appropriate folding and transportation pathways. Finally, insertion and anchoring of the protein to the plasma membrane could be facilitated by hydrophobic amino acids in the transmembrane domains of ATP1A4 (Homareda *et al*., 1989). Our study indicates the ability of sperm to synthesize protein suggests the importance of atypical, yet functional translation pathways to meet physiological demands during capacitation and opens interesting areas of investigation in mammalian sperm biology.

## ATP1A4 as a fertility marker in dairy bulls

In the Canadian Dairy Network (CDN), bull fertility is assessed by 56-day non-return rates (NRR), based on a linear mixed model adjusted for several factors, such as month of insemination, age of the cow or heifer at insemination, breed of service sire, price of inseminating dose, AI technician, and an overall herd management effect, all combined into one index (FERTSOL value). The bovine artificial insemination (AI) industry in Canada uses high-fertility (HF) and low-fertility (LF) Holstein bulls for breeding purposes. Based on this FERTSOL assessment, bulls were classified as either high-fertile (HF; FERTSOL > +1; range: 3.6-6.7) or low-fertile (LF; FERTSOL < -1; range: - 4 to -19).

Since mature sperm DNA is generally transcriptionally quiescent, sperm functions are carried out by existing proteins without additional protein synthesis (with few exceptions). Therefore, sperm proteins may serve as molecular markers for variations in bull fertility (Peddinti *et al*., 2008; D’Amours *et al*., 2010). We proposed that comparing sperm from bulls with varying levels of fertility may identify molecular differences (e.g. expression of specific proteins) in sperm and determine markers of fertility. Using frozen semen from HF and LF bulls as a research model, we investigated the role of ATP1A4 in regulation of fertility in dairy bulls.

Result of this study indicated that frozen-thawed sperm from HF bulls had increased ATP1A4 content and activity compared to LF bulls. A possible explanation for the difference in content between HF and LF bull sperm could be attributed either to loss of ATP1A4 from the sperm membrane of LF bulls during the process of freeze-thawing, or these bulls inherently might have had a lower content of ATP1A4. Unfortunately, it was not possible to investigate the latter possibility, due to a lack of access to fresh semen from these bulls.

Furthermore, post-thaw sperm from HF bulls had increased tyrosine phosphorylation, ROS, F-actin content, and low intracellular calcium concentrations compared to LF bulls. Subsequent incubation of HF sperm with ouabain further augmented post-thaw increases in tyrosine phosphorylation, ROS production, and F-actin content, whereas the increase in intracellular calcium was still low compared to LF sperm. Even though it is widely understood that increased ROS was related to cryo-capacitation (Bailey *et al*., 2000), results from our study indicate otherwise as HF sperm had low ROS content (Del Olmo *et al*., 2015). Perhaps there is a higher subpopulation of normal sperm (with a viable plasma membrane) compared to the cryo-capacitated sperm in HF and LF groups that is responsible for controlled ROS production after thawing. Furthermore, the higher level of ROS in HF sperm may correspond to the minimum threshold needed for successful capacitation (de Lamirande and Gagnon, 1995).

Calcium is another intracellular second messenger, with dynamic roles in hyperactivation and sperm capacitation. The interaction between Na/K- ATPase and inositol 1,4,5-triphosphate receptor (IP_3_R), an intracellular calcium store receptor, is regulated by ouabain, thereby increasing calcium concentrations. The presence of IP_3_ ligand and its cognate receptor, IP_3_R has already been confirmed in sperm (Ho and Suarez, 2003; Wennemuth *et al*., 2003). It is noteworthy that calcium increased in both fertility groups in response to ouabain, albeit at a higher magnitude in LF sperm. Like calcium, regulation of actin dynamics is pivotal for many sperm processes, including capacitation. Controlled production of ROS modulates reorganization of actin cytoskeletal components through a process that involves GTP binding protein Rho and actin binding protein cofilin (Carlier *et al*., 1999; Moldovan *et al*., 2000) or through involvement of gelsolin (Shahar *et al*., 2014). Therefore, it is likely that increased ROS could promote increased F-actin content in HF bull sperm.

Collectively, quantification of ATP1A4 and its associated downstream effectors (ROS, calcium or F- actin) may aid in development of improved laboratory assays for better prediction of fertility of bulls (Rajamanickam *et al.,* 2017b). This could prevent sub- fertile semen from entering the market and thereby improve efficiency of cattle reproduction.

## Conclusions and future directions

Using fresh and frozen-thawed bull semen, we demonstrated the physiological relevance of ATP1A4 in regulation of sperm function and its potential as a fertility marker. In the raft and non-raft study, we determined that raft and non-raft pools of ATP1A4 stimulated independent signaling pathways, leading to capacitation. Deducing downstream pathways mediated by ATP1A4 could help understand the molecular basis by which this protein functions in normal sperm, which in turn could help develop diagnostic approaches to identify male infertility. Mass spectrometry revealed interesting candidate proteins such as plakoglobin that interacted with ATP1A4 during capacitation. The functional significance of PLCζ as a sperm oocyte activating factor and the role of plakoglobin has been well studied in sperm and testis, respectively. However, the relevance of both these proteins interacting with ATP1A4 during capacitation and fertilization remains to be investigated.

Our results also indicated that there is ATP1A4 mRNA in capacitated sperm. Functionally intact and stable sperm mRNAs can be delivered to the oocyte during fertilization and are important during the critical window of activation of the embryonic genome and its epigenetic regulation (Sendler *et al*., 2013). Perhaps this sperm-specific mRNA persists in the oocyte until activation of the embryonic genome and has critical roles in embryo development. Further studies are expected to advance our current knowledge in the role of this protein in sperm function and fertilization.

## References

[B1] Ahmed NA, Salem MH, El-Oksh HA, Pursel VG. (1984). Effect of incubation conditions, inhibitors and seminal plasma on protein synthesis in ram spermatozoa. J Reprod Fertil.

[B2] Amikura R, Kashikawa M, Nakamura A, Kobayashi S. (2001). Presence of mitochondria-type ribosomes outside mitochondria in germ plasm of Drosophila embryos. Proc Natl Acad Sci U S A.

[B3] Amikura R, Sato K, Kobayashi S. (2005). Role of mitochondrial ribosome-dependent translation in germline formation in Drosophila embryos. Mech Dev.

[B4] Anpalakan K. (2010). Sodium, potassium-ATPase signalling mechanism inducing capacitation in bull sperm.

[B5] Arystarkhova E, Sweadner KJ. (1997). Tissue-specific expression of the Na,K-ATPase beta3 subunit. The presence of beta3 in lung and liver addresses the problem of the missing subunit. J Biol Chem.

[B6] Asano A, Selvaraj V, Buttke DE, Nelson JL, Green KM, Evans JE, Travis AJ. (2009). Biochemical characterization of membrane fractions in murine sperm: identification of three distinct sub-types of membrane rafts. J Cell Physiol.

[B7] Bailey JL, Bilodeau JF, Cormier N. (2000). Semen cryopreservation in domestic animals: a damaging and capacitating phenomenon. J Androl.

[B8] Bellin ME, Oyarzo JN, Hawkins HE, Zhang H, Smith RG, Forrest DW, Sprott LR, Ax RL. (1998). Fertility-associated antigen on bull sperm indicates fertility potential. J Anim Sci.

[B9] Blanco G, Mercer RW. (1998). Isozymes of the Na-K- ATPase: heterogeneity in structure, diversity in function. Am J Physiol.

[B10] Blanco G, Sanchez G, Melton RJ, Tourtellotte WG, Mercer RW. (2000). The alpha4 isoform of the Na,K- ATPase is expressed in the germ cells of the testes. J Histochem Cytochem.

[B11] Bou Khalil M, Chakrabandhu K, Xu H, Weerachatyanukul W, Buhr M, Berger T, Carmona E, Vuong N, Kumarathasan P, Wong PT, Carrier D, Tanphaichitr N. (2006). Sperm capacitation induces an increase in lipid rafts having zona pellucida binding ability and containing sulfogalactosylglycerolipid. Dev Biol.

[B12] Bragg PW, Handel MA. (1979). Protein synthesis in mouse spermatozoa. Biol Reprod.

[B13] Carlier MF, Ressad F, Pantaloni D. (1999). Control of actin dynamics in cell motility. Role of ADF/cofilin. J Biol Chem.

[B14] Christensen P, Hansen C, Liboriussen T, Lehn- Jensen H. (2005). Implementation of flow cytometry for quality control in four Danish bull studs. Anim Reprod Sci.

[B15] Cohen G, Rubinstein S, Gur Y, Breitbart H. (2004). Crosstalk between protein kinase A and C regulates phospholipase D and F-actin formation during sperm capacitation. Dev Biol.

[B16] Counillon L, Pouyssegur J. (2000). The expanding family of eucaryotic Na(+)/H(+) exchangers. J Biol Chem.

[B17] Cross NL. (2004). Reorganization of lipid rafts during capacitation of human sperm. Biol Reprod.

[B18] D'Amours O, Frenette G, Fortier M, Leclerc P, Sullivan R. (2010). Proteomic comparison of detergent- extracted sperm proteins from bulls with different fertility indexes. Reproduction.

[B19] Daniel L, Etkovitz N, Weiss SR, Rubinstein S, Ickowicz D, Breitbart H. (2010). Regulation of the sperm EGF receptor by ouabain leads to initiation of the acrosome reaction. Dev Biol.

[B20] Das PJ, McCarthy F, Vishnoi M, Paria N, Gresham C, Li G, Kachroo P, Sudderth AK, Teague S, Love CC, Varner DD, Chowdhary BP, Raudsepp T. (2013). Stallion sperm transcriptome comprises functionally coherent coding and regulatory RNAs as revealed by microarray analysis and RNA-seq. PloS one.

[B21] de Lamirande E, Gagnon C (1995). Impact of reactive oxygen species on spermatozoa: a balancing act between beneficial and detrimental effects. Hum Reprod.

[B22] de Lamirande E, O’Flaherty C (2008). Sperm activation: role of reactive oxygen species and kinases. Bichim Biophys Acta.

[B23] De Vries WN, Evsikov AV, Haac BE, Fancher KS, Holbrook AE, Kemler R, Solter D, Knowles BB (2004). Maternal beta-catenin and E-cadherin in mouse development. Development.

[B24] Del Olmo E, Bisbal A, Garcia-Alvarez O, Maroto- Morales A, Ramon M, Jimenez-Rabadan P, Anel- Lopez L, Soler AJ, Garde JJ, Fernandez-Santos MR. (2015). Free-radical production after post-thaw incubation of ram spermatozoa is related to decreased in vivo fertility. Reprod Fertil Dev.

[B25] Doris PA, Jenkins LA, Stocco DM. (1994). Is ouabain an authentic endogenous mammalian substance derived from the adrenal?. Hypertension.

[B26] Geering K. (1991). The functional role of the beta- subunit in the maturation and intracellular transport of Na,K-ATPase. FEBS letters.

[B27] Gilbert I, Bissonnette N, Boissonneault G, Vallee M, Robert C. (2007). A molecular analysis of the population of mRNA in bovine spermatozoa. Reproduction.

[B28] Gur Y, Breitbart H. (2006). Mammalian sperm translate nuclear-encoded proteins by mitochondrial-type ribosomes. Genes Dev.

[B29] Hansen GR. (2006). Animal Science Department.

[B30] Hickey KD, Buhr MM. (2011). Lipid bilayer composition affects transmembrane protein orientation and function. J Lipids.

[B31] Hlivko JT, Chakraborty S, Hlivko TJ, Sengupta A, James PF. (2006). The human Na,K-ATPase alpha 4 isoform is a ouabain-sensitive alpha isoform that is expressed in sperm. Mol Reprod Dev.

[B32] Homareda H, Kawakami K, Nagano K, Matsui H. (1989). Location of signal sequences for membrane insertion of the Na+, K + -ATPase alpha subunit. Mol Cell Biol.

[B33] Ho HC, Suarez SS. (2003). Characterization of the intracellular calcium store at the base of the sperm flagellum that regulates hyperactivated motility. Biol Reprod.

[B34] Jimenez T, Sanchez G, Wertheimer E, Blanco G. (2010). Activity of the Na,K-ATPase alpha4 isoform is important for membrane potential, intracellular Ca2+, and pH to maintain motility in rat spermatozoa. Reproduction.

[B35] Jimenez T, McDermott JP, Sanchez G, Blanco G. (2011a). Na,K-ATPase alpha4 isoform is essential for sperm fertility. Proc Natl Acad Sci U S A.

[B36] Jimenez T, Sanchez G, McDermott JP, Nguyen AN, Kumar TR, Blanco G. (2011b). Increased expression of the Na,K-ATPase alpha4 isoform enhances sperm motility in transgenic mice. Biol Reprod.

[B37] Jimenez T, Sanchez G, Blanco G. (2012). Activity of the Na,K-ATPase alpha4 isoform is regulated during sperm capacitation to support sperm motility. J Androl.

[B38] Jorgensen PL, Hakansson KO, Karlish SJ. (2003). Structure and mechanism of Na,K-ATPase: functional sites and their interactions. Annu Revf Physiol.

[B39] Kasimanickam V, Kasimanickam R, Arangasamy A, Saberivand A, Stevenson JS, Kastelic JP. (2012). Association between mRNA abundance of functional sperm function proteins and fertility of Holstein bulls. Theriogenology.

[B40] Kastelic JP, Thundathil JC. (2008). Breeding soundness evaluation and semen analysis for predicting bull fertility. Reprod Domest Anim.

[B41] Kumar G, Patel D, Naz RK. (1993). c-MYC mRNA is present in human sperm cells. Cell Mol Biol Res.

[B42] Kuster CE, Hess RA, Althouse GC. (2004). Immunofluorescence reveals ubiquitination of retained distal cytoplasmic droplets on ejaculated porcine spermatozoa. J Androl.

[B43] Liang M, Tian J, Liu L, Pierre S, Liu J, Shapiro J, Xie ZJ. (2007). Identification of a pool of non-pumping Na/K-ATPase. J Biol Chem.

[B44] Liu J, Tian J, Haas M, Shapiro JI, Askari A, Xie Z. (2000). Ouabain interaction with cardiac Na+/K+-ATPase initiates signal cascades independent of changes in intracellular Na+ and Ca2+ concentrations. J Biol Chem.

[B45] Liu L, Mohammadi K, Aynafshar B, Wang H, Li D, Liu J, Ivanov AV, Xie Z, Askari A. (2003). Role of caveolae in signal-transducing function of cardiac Na+/K+-ATPase. Am J Physiol Cell Physiol.

[B46] Mobasheri A, Avila J, Cozar-Castellano I, Brownleader MD, Trevan M, Francis MJ, Lamb JF, Martin-Vasallo P. (2000). Na+, K+-ATPase isozyme diversity; comparative biochemistry and physiological implications of novel functional interactions. Biosci Rep.

[B47] Moldovan L, Moldovan NI, Sohn RH, Parikh SA, Goldschmidt-Clermont PJ (2000). Redox changes of cultured endothelial cells and actin dynamics. Circ Res.

[B48] Naz RK. (1998). Effect of actinomycin D and cycloheximide on human sperm function. Arch Androl.

[B49] Newton LD, Kastelic JP, Wong B, van der Hoorn F, Thundathil J. (2009). Elevated testicular temperature modulates expression patterns of sperm proteins in Holstein bulls. Mol Reprod Dev.

[B50] Newton LD, Krishnakumar S, Menon AG, Kastelic JP, van der Hoorn FA, Thundathil JC. (2010). Na+/K+ATPase regulates sperm capacitation through a mechanism involving kinases and redistribution of its testis-specific isoform. Mol Reprod Dev.

[B51] Ninomiya Y, Ichinose S. (2007). Subcellular distribution of mitochondrial ribosomal RNA in the mouse oocyte and zygote. PloS one.

[B52] Nixon B, Aitken RJ. (2009). The biological significance of detergent-resistant membranes in spermatozoa. J Reprod Immunol.

[B53] O'Flaherty C, de Lamirande E, Gagnon C. (2006). Positive role of reactive oxygen species in mammalian sperm capacitation: triggering and modulation of phosphorylation events. Free Radic Biol Med.

[B54] Ostermeier GC, Dix DJ, Miller D, Khatri P, Krawetz SA. (2002). Spermatozoal RNA profiles of normal fertile men. Lancet.

[B55] Parent S, Lefièvre L, Brindle Y, Sullivan R. (1999). Bull subfertility is associated with low levels of a sperm membrane antigen. Mol Reprod Dev.

[B56] Peddinti D, Nanduri B, Kaya A, Feugang JM, Burgess SC, Memili E. (2008). Comprehensive proteomic analysis of bovine spermatozoa of varying fertility rates and identification of biomarkers associated with fertility. BMC Syst Biol.

[B57] Purdy PH. (2008). Ubiquitination and its influence in boar sperm physiology and cryopreservation. Theriogenology.

[B58] Rajamanickam GD, Kastelic JP, Thundathil JC. (2017a). Na/K-ATPase regulates bovine sperm capacitation through raft- and non-raft-mediated signaling mechanisms. Mol Reprod Dev.

[B59] Rajamanickam GD, Kroetsch T, Kastelic JP, Thundathil JC. (2017b). Testis-specific isoform of Na/K-ATPase (ATP1A4) regulates sperm function and fertility in dairy bulls through potential mechanisms involving reactive oxygen species, calcium and actin polymerization. Andrology.

[B60] Rajamanickam GD, Kastelic JP, Thundathil JC. (2017c). Content of testis-specific isoform of Na/K- ATPase (ATP1A4) is increased during bovine sperm capacitation through translation in mitochondrial ribosomes. Cell Tissue Res.

[B61] Rufas O, Fisch B, Ziv S, Shalgi R. (2000). Expression of cadherin adhesion molecules on human gametes. Mol Hum Reprod.

[B62] Schiefelbein D. (1998). Back to the Basics: A Real World Strategy for Improving the Quality and Consistency of Beef. In: Proceedings of the Annual Mtg. 30th of Beef Improvement Federation, Calgary, AB.

[B63] Sendler E, Johnson GD, Mao S, Goodrich RJ, Diamond MP, Hauser R, Krawetz SA. (2013). Stability, delivery and functions of human sperm RNAs at fertilization. Nucleic Acids Res.

[B64] Shadan S, James PS, Howes EA, Jones R. (2004). Cholesterol efflux alters lipid raft stability and distribution during capacitation of boar spermatozoa. Biol Reprod.

[B65] Shahar S, Hillman P, Lubart R, Ickowicz D, Breitbart H. (2014). Activation of sperm EGFR by light irradiation is mediated by reactive oxygen species. Photochem Photobiol.

[B66] Skou JC, Esmann M. (1992). Chapter One: The Na, K- ATPase. J Bioenerg Biomembr.

[B67] Sleight SB, Miranda PV, Plaskett NW, Maier B, Lysiak J, Scrable H, Herr JC, Visconti PE. (2005). Isolation and proteomic analysis of mouse sperm detergent-resistant membrane fractions: evidence for dissociation of lipid rafts during capacitation. Biol Reprod.

[B68] Sutovsky P, Terada Y, Schatten G. (2001). Ubiquitin- based sperm assay for the diagnosis of male factor infertility. Hum Reprod.

[B69] Sutovsky P, Neuber E, Schatten G. (2002). Ubiquitin- dependent sperm quality control mechanism recognizes spermatozoa with DNA defects as revealed by dual ubiquitin-TUNEL assay. Mol Reprod Dev.

[B70] Sutovsky P, Turner RM, Hameed S, Sutovsky M. (2003). Differential ubiquitination of stallion sperm proteins: possible implications for infertility and reproductive seasonality. Biol Reprod.

[B71] Sutovsky P, Aarabi M, Miranda-Vizuete A, Oko R. (2015). Negative biomarker based male fertility evaluation: Sperm phenotypes associated with molecular-level anomalies. Asian J Androl.

[B72] Takezawa Y, Yoshida K, Miyado K, Sato M, Nakamura A, Kawano N, Sakakibara K, Kondo T, Harada Y, Ohnami N, Kanai S, Miyado M, Saito H, Takahashi Y, Akutsu H, Umezawa A. (2011). Beta- Catenin is a Molecular Switch that Regulates Transition of Cell-Cell Adhesion to Fusion. Sci Rep.

[B73] Thundathil JC, Anzar M, Buhr MM. (2006). Na+/K+ATPase as a signaling molecule during bovine sperm capacitation. Biol Reprod.

[B74] Toshimori K. (2009). Dynamics of the sperm head: Modification and maturation events from spermatogenesis to egg activation. Adv Anat Embryol Cell Biol.

[B75] van Gestel RA, Brewis IA, Ashton PR, Helms JB, Brouwers JF, Gadella BM. (2005). Capacitation- dependent concentration of lipid rafts in the apical ridge head area of porcine sperm cells. Mol Hum Reprod.

[B76] van Gestel RA, Brewis IA, Ashton PR, Brouwers JF, Gadella BM. (2007). Multiple proteins present in purified porcine sperm apical plasma membranes interact with the zona pellucida of the oocyte. Mol Hum Reprod.

[B77] Villegas J, Araya P, Bustos-Obregon E, Burzio LO. (2002). Localization of the 16S mitochondrial rRNA in the nucleus of mammalian spermatogenic cells. Mol Hum Reprod.

[B78] Wagoner K, Sanchez G, Nguyen AN, Enders GC, Blanco G. (2005). Different expression and activity of the alpha1 and alpha4 isoforms of the Na,K-ATPase during rat male germ cell ontogeny. Reproduction.

[B79] Wennemuth G, Babcock DF, Hille B. (2003). Calcium clearance mechanisms of mouse sperm. J Gen Physiol.

[B80] Woo AL, James PF, Lingrel JB. (2000). Sperm motility is dependent on a unique isoform of the Na,K-ATPase. J Biol Chem.

[B81] Woo AL, James PF, Lingrel JB. (2002). Roles of the Na,K-ATPase alpha4 isoform and the Na+/H+ exchanger in sperm motility. Mol Rprod Dev.

[B82] Xie Z, Kometiani P, Liu J, Li J, Shapiro JI, Askari A. (1999). Intracellular reactive oxygen species mediate the linkage of Na+/K+-ATPase to hypertrophy and its marker genes in cardiac myocytes. J Biol Chem.

[B83] Yagi A, Paranko J. (1995). Actin, alpha-actinin, and spectrin with specific associations with the postacrosomal and acrosomal domains of bovine spermatozoa. Anat Rec.

[B84] Yamada S, Pokutta S, Drees F, Weis WI, Nelson WJ. (2005). Deconstructing the cadherin-catenin-actin complex. Cell.

